# Nitric Oxide Synthase 1 Modulates Basal and β-Adrenergic-Stimulated Contractility by Rapid and Reversible Redox-Dependent S-Nitrosylation of the Heart

**DOI:** 10.1371/journal.pone.0160813

**Published:** 2016-08-16

**Authors:** Alejandra Z. Vielma, Luisa León, Ignacio C. Fernández, Daniel R. González, Mauricio P. Boric

**Affiliations:** 1 Departamento de Fisiología, Facultad de Ciencias Biológicas, Pontificia Universidad Católica de Chile, PO Box 114-D, Santiago, Chile; 2 Departamento de Ciencias Básicas Biomédicas, Facultad de Ciencias de la Salud, Universidad de Talca, Av. Lircay S.N., Talca, Chile; Newcastle University, AUSTRALIA

## Abstract

S-nitrosylation of several Ca^2+^ regulating proteins in response to β-adrenergic stimulation was recently described in the heart; however the specific nitric oxide synthase (NOS) isoform and signaling pathways responsible for this modification have not been elucidated. NOS-1 activity increases inotropism, therefore, we tested whether β-adrenergic stimulation induces NOS-1-dependent S-nitrosylation of total proteins, the ryanodine receptor (RyR2), SERCA2 and the L-Type Ca^2+^ channel (LTCC). In the isolated rat heart, isoproterenol (10 nM, 3-min) increased S-nitrosylation of total cardiac proteins (+46±14%) and RyR2 (+146±77%), without affecting S-nitrosylation of SERCA2 and LTCC. Selective NOS-1 blockade with S-methyl-L-thiocitrulline (SMTC) and N^ω^-propyl-l-arginine decreased basal contractility and relaxation (−25–30%) and basal S-nitrosylation of total proteins (−25–60%), RyR2, SERCA2 and LTCC (−60–75%). NOS-1 inhibition reduced (−25–40%) the inotropic response and protein S-nitrosylation induced by isoproterenol, particularly that of RyR2 (−85±7%). Tempol, a superoxide scavenger, mimicked the effects of NOS-1 inhibition on inotropism and protein S-nitrosylation; whereas selective NOS-3 inhibitor L-N^5^-(1-Iminoethyl)ornithine had no effect. Inhibition of NOS-1 did not affect phospholamban phosphorylation, but reduced its oligomerization. Attenuation of contractility was abolished by PKA blockade and unaffected by guanylate cyclase inhibition. Additionally, in isolated mouse cardiomyocytes, NOS-1 inhibition or removal reduced the Ca^2+^-transient amplitude and sarcomere shortening induced by isoproterenol or by direct PKA activation. We conclude that 1) normal cardiac performance requires basal NOS-1 activity and S-nitrosylation of the calcium-cycling machinery; 2) β-adrenergic stimulation induces rapid and reversible NOS-1 dependent, PKA and ROS-dependent, S-nitrosylation of RyR2 and other proteins, accounting for about one third of its inotropic effect.

## Introduction

The heart responds rapidly when the organism is challenged by physical or psychological stress, increasing cardiac output (fight or flight response). In these situations, the sympathetic branch of the autonomic system releases catecholamines (adrenaline and noradrenaline) and elicits this response in the heart by acting on β adrenergic receptors. Upon catecholamine binding, β adrenergic receptors, which are G-protein coupled receptors, activate adenylate cyclase to generate the second messenger cAMP, which in turn, activates protein kinase A (PKA). Ultimately, PKA phosphorylates several proteins in the cardiac myocyte that are responsible for increasing contractility and lusitropy (the ability to relax rapidly during diastole). Most of these proteins participate in the process of excitation-contraction coupling, namely the L-type calcium channel (LTCC), phospholamban (PLB) and the ryanodine receptor RyR2, in addition to proteins of the myofilaments. The phosphorylation of these proteins ultimately leads to an increase in the amplitude of the calcium transients that generate each heartbeat, increasing heart force and accelerating relaxation [1]

Interestingly, in recent years it has been described that this adrenergic response in the heart is modulated by the gasotransmitter nitric oxide (NO). In addition to the classic effects mediated by the generation of cyclic guanosine monophosphate (cGMP) and the activation of protein kinase G (PKG), NO exerts ubiquitous signaling via post-translational modification of thiol groups of specific cysteine residues, a reaction termed S-nitrosylation [2], depending on the redox environment [3]. In the heart, important substrates of S-nitrosylation that may influence cardiac function include receptors, enzymes, ion channels, transcription factors, and structural proteins [4, 5]. Cardiac proteins involved in the excitation-contraction coupling process are also potentially regulated by S-nitrosylation [6, 7]. Recently it was reported that β-adrenergic stimulation causes S-nitrosylation of several proteins involved in Ca^2+^ cycling, notably troponin-C, phospholamban (PLB) and sodium-calcium exchanger (NCX) [8]. However, the specific NOS isoform responsible for this modification has not been reported.

In the cardiomyocyte, NO production is tightly compartmentalized by differential subcellular location of the nitric oxide synthase isoforms [6, 9]: neuronal nitric oxide synthase 1 (NOS-1) is located in the sarcoplasmic reticulum (SR) [10], while endothelial NOS (NOS-3) is located to plasmalemmal caveolae [11]. Inducible NOS (NOS-2) is expressed mainly in the failing heart, as a cytosolic protein [12]. Furthermore, the activity of both constitutive NOS isoforms has been associated with opposing inotropic effects, based on the observation that NOS-1 deficiency impairs inotropism [13] while NOS-3 deficiency exacerbates it [13, 14]. It has been postulated that NOS-3 negatively regulates the L-type calcium current [14], likely through the activation of the cGMP-PKG pathway [15, 16]; while NOS-1 may directly affect the calcium cycling machinery through S-nitrosylation of the cardiac ryanodine receptor (RyR2) [17, 18]. Nevertheless, the mechanisms by which NOS-1 facilitates cardiac contractility remain controversial [18–20]. Although NOS-1 has been reported to influence cardiac contractility through S-nitrosylation, it is unknown whether this modification is involved in acute modulation of heart function, such as that elicited by adrenergic stimulation. Recently it was reported that NOS-1 activity partakes in β-adrenergic signaling activating CaMKII, which leads to increased SR Ca^2+^ leak through RyR2 [21]. Thus, we tested the hypothesis that β-adrenergic stimulation induces NOS-1-dependent S-nitrosylation of cardiac proteins, particularly those involved in the Ca^2+^ cycle handling, and that this modification favors contractility.

## Materials and Methods

### Animals

Male Sprague—Dawley rats (290–310g) were obtained from the animal facility of the Facultad de Ciencias Biológicas, Pontificia Universidad Católica de Chile (FCB-PUC, Santiago, Chile). Male C57Bl/6 and eNOS KO mice were purchased from Jackson Laboratories (Bar Harbor, MA). All the experimental protocols were approved by the respective Committee of Bioethics and Bio-safety of the FCB-PUC, and the Facultad de Ciencias de la Salud, Universidad de Talca, and conformed to the Guide for the Care and Use of Laboratory Animals published by the US National Institutes of Health (NIH publication No. 85–23, revised 1996).

### Isolated heart preparation

Rats were anesthetized with ketamine (90mg/Kg) and xylazine (10mg/Kg) and the hearts were excised and perfused with Krebs-Henseleit buffer in as described [22]. The Langendorff preparation was perfused at a constant flow (10mL/min) to avoid the Gregg effect; therefore, changes in perfusion pressure represent changes in coronary vascular resistance. Hearts were paced at 5 Hz, to prevent the influence of chronotropic effects. After an equilibration period (15-min) all hearts were stimulated with a 3-min pulse of 10nM isoproterenol, followed by a 10-min recovery period. This initial stimulation sequence served to assess basal contractility and the inotropic response to isoproterenol at a concentration close to the EC_50_ observed in our conditions.

We built accumulative concentration-response curves to isoproterenol in the presence or absence of NOS-1 inhibitors S-methyl-L-thiocitrulline (SMTC, 300nM) [23, 24] and N^ω^-propyl-l-arginine (LNPA, 200nM) [25], the preferential NOS-3 inhibitor L-N5-(1-Iminoethyl)ornithine (LNIO, 1μM) [26, 27], the thiyl-radical scavenger tempol (100μM) [28], which inhibits the process of S-nitrosylation both in vitro and in biological systems [22, 29]; or a combination of SMTC plus tempol. Hearts were perfused with the inhibitor, or with vehicle during 20-min and stimulated successively with increasing concentrations of isoproterenol (0.1nM -1.0μM, 3-min each) in the presence of the inhibitor.

Further experiments were performed to assess the involvement of PKA and PKG signaling pathways using the following abridged protocols. First, to assess PKA, after equilibration, we performed a cumulative concentration response to isoproterenol (1-100nM), followed by 20-min perfusion with PKA inhibitor N-[2-[[3-(4-Bromophenyl)-2-propenyl]amino]ethyl]-5-isoquinolinesulfonamide (H89) Tochris, Bristol, UK(0.5μM) [30] either alone, or combined with SMTC (300nM), prior to a second isoproterenol concentration-response curve in the presence of the inhibitors. Second, to assess involvement of the guanylate cyclase-PKG pathway, we compared the response to isoproterenol in control conditions, and after inhibiting NOS1 in two conditions: when PKG activation was suppressed by guanylate cyclase inhibitor 1H-[1,2,4]Oxadiazolo[4,3-a]quinoxalin-1-one ODQ (10μM) (Tochris, Bristol, UK) [22], or when PKG activation was held constant by adding ODQ (10μM) combined with 1μM 8Br-cGMP, a cell-permeable cGMP analog.

To confirm the involvement of NOS-1 in the S-nitrosylation process, further experiments were performed on perfused hearts of wild type (WT) and NOS-3 deficient mice. Mice were anesthetized with ketamine-xylazine, and their hearts were perfused through the aorta at 4mL/min, following an abbreviated protocol compared with that used in rats: after 20-min stabilization, hearts were stimulated with 10nM Isoproterenol or vehicle for 3 min, and quickly harvested for biochemical analysis.

### Measurement of sarcomere shortening and intracellular Ca^2+^ concentration [Ca^2+^]_i_

Cardiac myocytes were isolated from mice as previously described [17]. The myocytes were loaded with 1μM fura-2/AM (15-min), and transferred to a Lucite chamber with a glass coverslip on the stage of an inverted microscope (NIKON Eclipse T*i*-S). Cells were continuously superfused in Tyrode and field-stimulated using platinum electrodes. Isoproterenol was added to the superfusion; SMTC was added to the bath 20 min prior to measurements and included in the bathing solutions throughout. Two stimulation conditions were utilized: initially, full isoproterenol concentration response curves were obtained using low frequency (1-Hz) and high extracellular Ca^2+^ (1.8mM) at room temperature; then we analyzed single concentration responses to isoproterenol (10nM) in 1.2mM Ca^2+^ at 3-Hz, 37°C. Further experiments were conducted to test for maximal PKA activation using a soluble cyclic AMP analog Db-cAMP 1mM, in WT and NOS-1 deficient mice (Jackson Laboratories). Sarcomere length was recorded in real time using an iCCD camera and specialized data acquisition software (IonWizard SarcLen Acquisition System, IonOptix, Inc). Twitch amplitude was expressed as the ratio of sarcomere shortening (difference between peak systolic and diastolic length) to diastolic length [17, 31]. [Ca^2+^]_i_ was determined by the fura-2- 360/380nm fluorescence ratio using a dual-excitation spectrofluorometer (IonOptix, MA), as described [17, 31].

### Detection of S-nitrosylated proteins

Isolated rat hearts were submitted to the initial control stimulation sequence, then perfused with an inhibitor or its vehicle for 20-min and either stimulated with isoproterenol 10nM for 3-min (stimulated) or just perfused for additional 3-min (basal). The heart was quickly removed and a piece of ventricular tissue (~200mg) was homogenized in 1mL of cold Tris-HCl (100mM, pH 7.4) containing protease inhibitors.

A 200μg protein sample of each tissue homogenate was treated to detect S-nitrosylated proteins by the biotin-switch method [32, 33]. First, free thiols (-SH) were blocked for 1-h at 50°C in the dark with methyl methanethiosulfonate (MMTS). Proteins were precipitated with 4 volumes of ice-cold acetone, washed repeatedly with acetone to remove free MMTS and resolubilized. Thereafter, nitrosylated cysteine residues (-S-NO) were reduced to free cysteine by incubating 1-hour with 30mM sodium ascorbate and selectively labeled with HPDP-biotin. The proteins were precipitated again with acetone to wash the excess of HPDP-biotin and solubilized for Western blot analysis. As a negative control, some samples were not treated with ascorbate, the agent required to reduce S-NO groups prior to biotin labeling.

#### General proteins

Proteins were resolved in a 7.5% SDS-PAGE, electroblotted to a nitrocellulose membrane and stained with Ponceau red to evaluate the total protein content. Then, the biotinylated proteins were visualized using an anti-biotin antibody conjugated to peroxidase (Cell Signaling, Beverly, MA, USA).

#### Specific proteins

Following solubilization, the samples were incubated 1-hour with agarose-conjugated streptavidin beads and centrifuged to pull-down HPDP-biotinylated proteins. Adsorbed proteins were eluted with reducing buffer and separated by Nu-PAGE 3–8% Tris-Acetate gel electrophoresis (Invitrogen, Grand Island, NY), blotted on nitrocellulose, and analyzed using the following antibodies: monoclonal anti-RyR2 (Affinity Bioreagents, Golden, CO), goat polyclonal anti-SERCA2 (Santa Cruz Biotechnology, Santa Cruz, CA), and monoclonal anti-calcium channel L-type DHPR alpha-2 subunit (Abcam Plc, Cambridge, MA). For all Western blot analysis, the intensity of the signal was evaluated using the Image J program (NIH public domain software).

In addition, the presence and phosphorylation of proteins were detected by regular Western blots, using these primary antibodies: PLB, phospho-Ser16-PBL, and phospho-Thr17-PLB from Badrilla (Leeds, UK); NOS-2 (iNOS) and NOS-3 (nNOS) from Santa Cruz (Dallas, TX); NOS-1 and phospho-Ser-1412-NOS-1 from Abcam Plc (Cambridge, MA).

### Statistical Analysis

Data are presented as means ± SEM. Data from concentration-response experiments were fitted to a sigmoid pharmacological equation obtaining minimum, maximum and EC_50_ values. Differences between curves were analyzed by two-way ANOVA, using the Newman-Keuls post-hoc test. Single concentration effects between two treatments were compared by Student’s t test, either paired or unpaired, and applying Dunnett’s modification for multiple comparisons against a single control, as appropriate. Two-way ANOVA was used when analyzing the effects of treatment (inhibitors) and stimulation (isoproterenol). The Graphpad Prism 4.0 software (Graphpad Software Inc, San Diego, CA, USA) was used for these analyses.

## Results

### Effect of NOS-1 activity on cardiac contractility

First, we assessed the importance of NOS-1 in the function of the isolated rat heart by pharmacological inhibition of the enzyme. For this, we used two pharmacological inhibitors, SMTC and LNPA, which are reported to have respectively one and two orders of magnitude higher affinity for NOS-1 than for NOS-3, the two main isoforms expressed in the heart [34]. Minimal impact on NOS-3 is expected since SMTC and LNPA were used at concentrations corresponding to approximately 1:20 and 1:40 of the respective IC_50_ value for that isoform [23, 25]. Therefore, we are confident that at the concentrations used, SMTC and LNPA have preferentially inhibited NOS-1.

**Fig 1:**
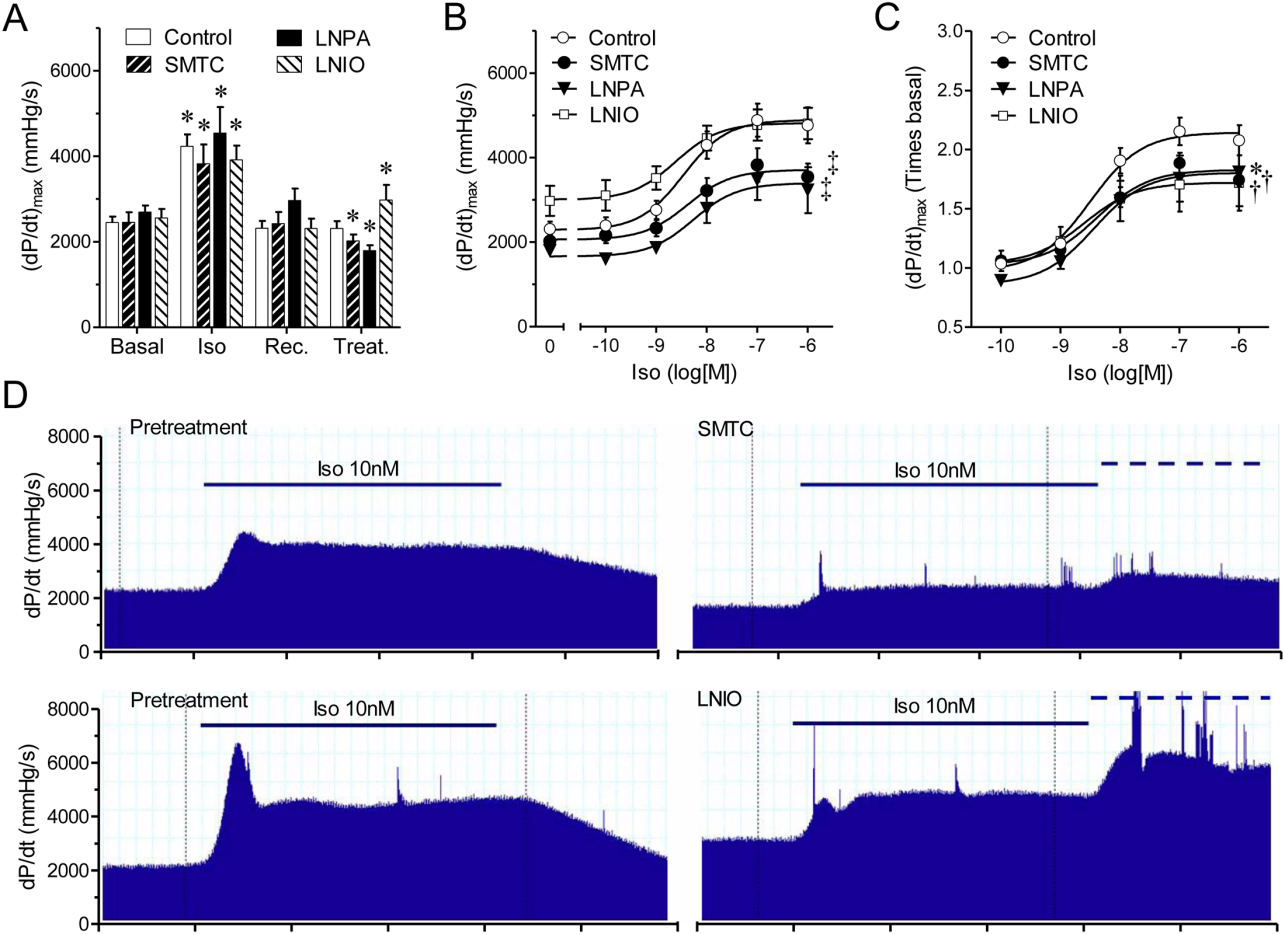
NOS-1 inhibition reduces the inotropic response to β-adrenergic stimulation. **(A)** Contractility (dP/dt_max_) values obtained consecutively under basal conditions (Basal), stimulation with isoproterenol (10nM, 3-min) (Iso); recovery after 10-min washout of the agonist (Rec.), and after 20-min treatment (Treat.) with vehicle (Control, n = 14), or NOS-1 inhibitors L-Methyl-thio-citrulline 300nM (SMTC, n = 8), or N^ω^—propyl—L—arginine 200nM (LNPA, n = 6), or NOS-3 inhibitor L-N^5^-(1-Iminoethyl)ornithine 1μM (LNIO, n = 6). Asterisk indicates p<0.05 vs. respective Basal (paired t test, with Dunnett’s modification). **(B)** Accumulative concentration-response curves to isoproterenol in absence (Control) or presence of NOS-1 inhibitors SMTC or LNPA, or NOS-3 inhibitor LNIO (‡ p<0.001 vs. Control curve, best fit ANOVA, the difference resided only in the top value; bottom and EC_50_ parameters were shared). **(C)** Same data as in panel B, expressed as relative values, corrected by the corresponding contractility values after treatment with inhibitor. (* p<0.05 (LNPA), † p<0.01 (SMTC, LNIO) vs. control curve; the difference resided in the top value). **(D)** Representative records showing contractility (dP/dt) responses elicited by application of Iso10nM (solid line) before and after treatment with SMTC (top) and LNIO (bottom). The dashed line indicates application of 100nM Iso as part of the dose-response curve.

All hearts had similar basal contractility and responded similarly to the test dose of isoproterenol (Fig 1A). In control conditions, hearts exposed to increased concentrations of isoproterenol (0.1nM-1μM, 3-min each) displayed a sigmoid concentration-response curve (Fig 1B). Pre-treatment with SMTC (300nM, for 20-min) reduced this response, shifting the response curve downward by approximately one third (Fig 1B). Similar inhibition was observed with LNPA (200nM, 20-min). Both inhibitors caused significant reductions (~25–30%) in basal contractility (Fig 1A), developed pressure, and lusitropy, and increases in perfusion pressure (Table 1). Despite the decrease in basal contractility, a significant reduction in the inotropic response to isoproterenol was still observed in hearts treated with NOS-1 inhibitors as compared to control when the change in contractility was analyzed using values relative to the respective baseline after treatment (Fig 1C). Neither inhibitor modified the EC_50_ for isoproterenol.

**Table 1:** Hemodynamic parameters in isolated hearts.

Experimental Group	Pre-Treatment		Post-Treatment	
	Basal	Iso	Treatment	Iso
***CPP (mmHg)***				
**Control** (33)	62.1 ± 3.9	53.9 ± 3.4 **	67.0 ± 4.1	55.0 ± 3.7 (25) *** †
**SMTC** (24)	53.8 ± 2.8	48.2 ± 2.9 ***	80.9 ± 5.8 ‡	61.6 ± 7.9 (16) ** †
**LNPA** (15)	59.9 ± 5.3	56.0 ± 4.1 *	90.3 ± 7.7 †	74.9 ± 9.7 (10) * †
**LNIO** (22)	59.5 ± 3.5	59.0 ± 3.0	70.5 ± 5.7 †	65.8 ± 4.6 (14) ¬
**Tempol** (19)	48.3 ± 4.8	44.0 ± 2.9 *	71.5 ± 7.4 †	59.1 ± 5.6 (14) ** †
**Tempol + SMTC** (6)	48.0 ± 2.8	43.7 ± 2.5 **	67.3 ± 6.9 †	55.8 ± 8.5 (6) **
***LVDP (mmHg)***				
**Control** (33)	77.3 ± 4.3	109.8 ± 5.3 ***	65.4 ± 2.9 ‡	100.5 ± 5.8 (25) ***
**SMTC** (24)	82.1 ± 4.7	114.4 ± 6.3 ***	63.4 ± 3.2 ‡	85.8 ± 5.0 (16) † ***
**LNPA** (15)	72.0 ± 6.3	97.9 ± 5.2 ***	45.5 ± 3.2 ‡	70.5 ± 5.1 (10) † ***
**LNIO** (22)	64.0 ± 3.2	113.2 ± 8.4 ***	66.3 ± 4.0	100.1 ± 9.7 (14) ***
**Tempol** (19)	84.0 ± 6.2	114.1 ± 6.7 ***	59.9 ± 3.7 ‡	93.1 ± 6.7 (14) ¬ ***
**Tempol + SMTC** (6)	65.6 ± 6.2	114.4 ± 10.6 **	58.0 ± 5.1	93.3 ± 8.6 ¬ ***
***(dP/dt)***_***min***_ ***mmHg/s]***				
**Control** (33)	‒ 1711 ± 106	‒ 2848 ± 157 ***	‒ 1445 ± 86 †	‒ 2582 ± 154 (25) ***
**SMTC** (24)	‒ 1945 ± 117	‒ 2855 ± 183 ***	‒ 1340 ± 80 ‡	‒ 2098 ± 215 (16) *** ¬
**LNPA** (15)	‒ 1995 ± 199	‒ 2861 ± 147 ***	‒ 1132 ± 62 ‡	‒ 1864 ± 198 (10) *** ‡
**LNIO** (22)	‒ 1264 ± 104	‒ 2490 ± 145 ***	‒ 1283 ± 96	‒ 2357 ± 238 (14) ***
**Tempol** (19)	‒ 1895 ± 124	‒ 3106 ± 167 ***	‒ 1247 ± 76 ‡	‒ 1889 ± 182 (14) *** ‡
**Tempol + SMTC** (6)	‒ 1329 ± 66	‒ 2876 ± 312 **	‒ 1303 ± 78	‒ 2459 ± 338 (6) ** ¬
***(dP/dt)***_***max***_, ***(mmHg/s)***				
**Control** (33)	2592 ± 113	4170 ± 181 ***	2316 ± 111 †	4302 ± 288 (25) ***
**SMTC** (24)	2608 ± 114	4094 ± 188 ***	1889 ± 101 ‡	2806 ± 248 (16) † **
**LNPA** (15)	2771 ± 192	4495 ± 231 ***	1971 ± 132 †	2692 ± 334 (10) ‡ ***
**LNIO** (22)	2484 ± 116	4657 ± 292 ***	2770 ± 114 ¬	4812 ± 283 (11) ***
**Tempol** (19)	2788 ± 149	4132 ± 210 ***	1930 ± 118 ‡	2753 ± 219 (14) ‡ ***
**Tempol + SMTC** (6)	2144 ± 143	3934 ± 272 **	1989 ± 177	3119 ± 308 ¬ ***

Table 1 summarizes the values of Coronary Perfusion Pressure (CCP); Left Ventricular Developed Pressure (LVDP); Maximal rate of pressure relaxation ((dP/dt)_min_); and Maximal rate of pressure development ((dP/dt)_max_), for all hearts included in this study. In all conditions, heart rate was kept constant, at 300 BPM. The first 2 columns (Pre-Treatment) show values at the end of a 20-min equilibration period (Basal) and after a 3-min stimulus with 10nM Isoproterenol (Iso). Hearts were allowed a 10-min period of washout, continued with 20-min perfusion with one inhibitor, or a combination of inhibitors, or vehicle, as indicated in the Experimental Group category (Post-Treatment), which was followed by a second challenge with 10nM Isoproterenol in the presence of the inhibitor (Iso). The number of hearts in each group is shown in parenthesis, next to the group’s name for columns 1–3, or within the fourth column. The fourth column has lower numbers because some hearts were homogenized for biochemical studies before the second stimulation.

* = p<0.05

** = p<0.001

*** = p<0.0001

Iso vs. precedent basal value, (paired t test)

^¬^ = p<0.05

^†^ = p<0.01

^‡^ = p<0.001 vs. respective Pre-Treatment value (paired t test)

Our observations that both SMTC and LNPA increased coronary perfusion pressure confirmed that NOS-1 influences coronary blood flow [35]. This opens the possibility that perfusion changes contribute to the observed effects on cardiac contractility. We addressed this concern in two ways. First, we used LNIO, a blocker with preferential selectivity for NOS-3, at a concentration well below the reported IC_50_ value for NOS-1. Application of LNIO (1μM, 20-min), increased perfusion pressure to a similar extent as SMTC or LNPA (Table 1), however, contrary to NOS-1 inhibitors, LNIO increased basal contractility (Fig 1A) and did not modify the inotropic response to isoproterenol in absolute values (Fig 1B). Because of the increased baseline in hearts treated with LNIO, the maximal relative increase in contractility was smaller than control (Fig 1C).

**Fig 2:**
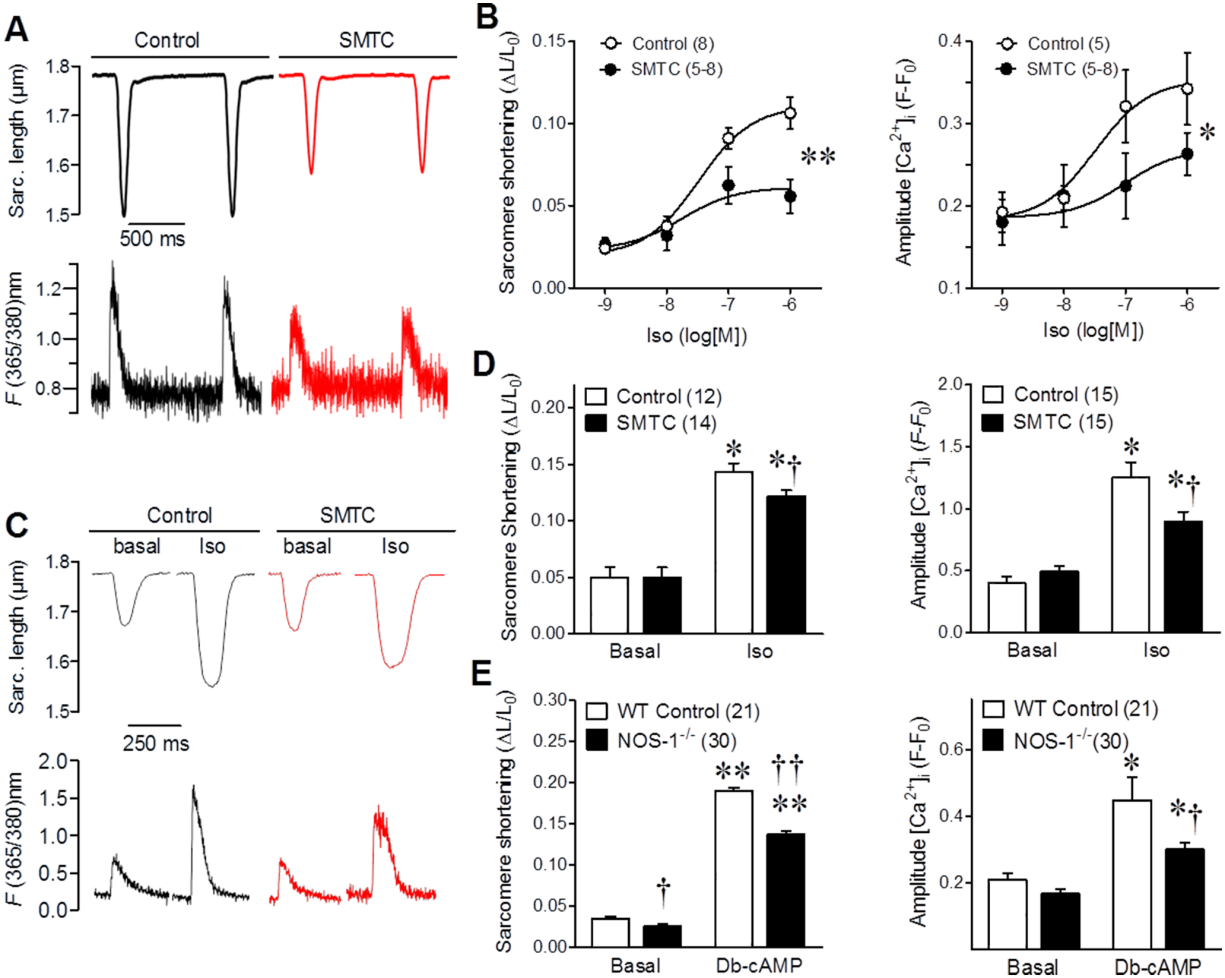
NOS-1 inhibition reduces contractility and calcium transients in response to β-adrenergic stimulation in isolated cardiomyocytes. **(A)** Freshly isolated mouse cardiomyocytes were incubated in 1.8 mM extracellular Ca^2+^ and field stimulated at 1 Hz. Representative traces of electrically-evoked sarcomere shortening (upper traces) and calcium transients (lower traces) in myocytes challenged with 1μM isoproterenol, in the presence or absence of L-Methyl-thio-citrulline 300nM (SMTC). **(B)** Graphs depict concentration-response curves of the degree of sarcomere shortening, expressed as fraction of resting length (ΔL/L_0_) in response to isoproterenol (**Left**) and the amplitude of the [Ca^2+^]_i_ increase from baseline (fractional change in fluorescence intensity (**Right**), in control or SMTC treated cardiomyocytes. The number of myocytes in each curve point is shown in parenthesis, coming from 5 different hearts each group. * p<0.05; ** p<0.01 vs. control curve (2-way ANOVA). **(C)** Myocytes were incubated in 1.2 mM extracellular Ca^2+^ and field stimulated at 3 Hz. Representative traces of electrically-evoked sarcomere shortening (upper traces) and calcium transients (lower traces) in basal conditions or in 1μM isoproterenol, in the presence or absence of SMTC 300nM. **(D)** Bar graphs depict the average sarcomere shortening (**Left**) and amplitude of the [Ca^2+^]_i_ increase (**Right**) for the number of myocytes mentioned in the header, from 5 hearts per group. **(E)** Myocytes obtained from wild type and NOS-1 null mice were field stimulated at 1 Hz, in 1.8 mM extracellular Ca^2+^ in control conditions or after adding 1 mM Db-cAMP to produce full PKA activation. Average sarcomere shortening (**Left**) and amplitude of the [Ca^2+^]_i_ increase (**Right**), in parenthesis number of myocytes obtained from 3 different hearts each group. * p<0.05, ** p<0.001 vs. basal; † p<0.05, †† p<0.001 vs. control (t test).

Secondly, to further rule out possible influences of vascular changes in the observed effects in cardiac contractility, we performed experiments in isolated cardiomyocytes. For this, we obtained isolated myocytes from mice, a well characterized model used to study excitation-contraction coupling. The mouse is a species that shares several electrophysiological features with the rat [36], particularly the Sprague-Dawley rats is akin with C57Bl6 mice [37], making the observations comparable. First, we challenged myocytes with increasing concentrations of isoproterenol in the presence or absence of SMTC. Both, the degree of sarcomere shortening and the amplitude of the evoked calcium transients ([Ca^2+^]_i_) were significantly attenuated by NOS-1 inhibition (Fig 2A and 2B). Concomitantly, the time constant for [Ca^2+^]_i_ decay was increased by SMTC (in ms: 114±13, 112±16, 102±20, and 87±10, at 1nM,10nM, 100nM and 1μM Iso, vs. respective control values of 100±7, 95±10, 79±10 and 59±5, p<0.05). Although these stimulation conditions have been used extensively for this kind of experiments; there are chances that deviation from in vivo conditions may influence the results. Therefore, we analyzed the response to 1μM isoproterenol in cardiomyocytes under more physiological conditions (3Hz, 1.2mM Ca^2+^, at 37°C); confirming that calcium transient and cell contraction were significantly attenuated in the presence of 300nM SMTC (Fig 2C and 2D). These results corroborate the cardiomyocyte origin of the reduced contractility observed after NOS-1 inhibition, confirming that lack of NOS-1 activity causes a reduced inotropic response to β-adrenergic stimulation [13, 38]. To further confirm the role of NOS-1 in this effect, we used NOS-1null cardiomyocytes, this time activated with exogenous cAMP, in order to directly activate PKA. As seen in Fig 2E, NOS-1null cardiomyocytes presented a ~30% smaller response to Db-cAMP administration as compared to wild type myocytes.

### Effect of isoproterenol on S-nitrosylation and its dependency on NOS-1 activity

**Fig 3:**
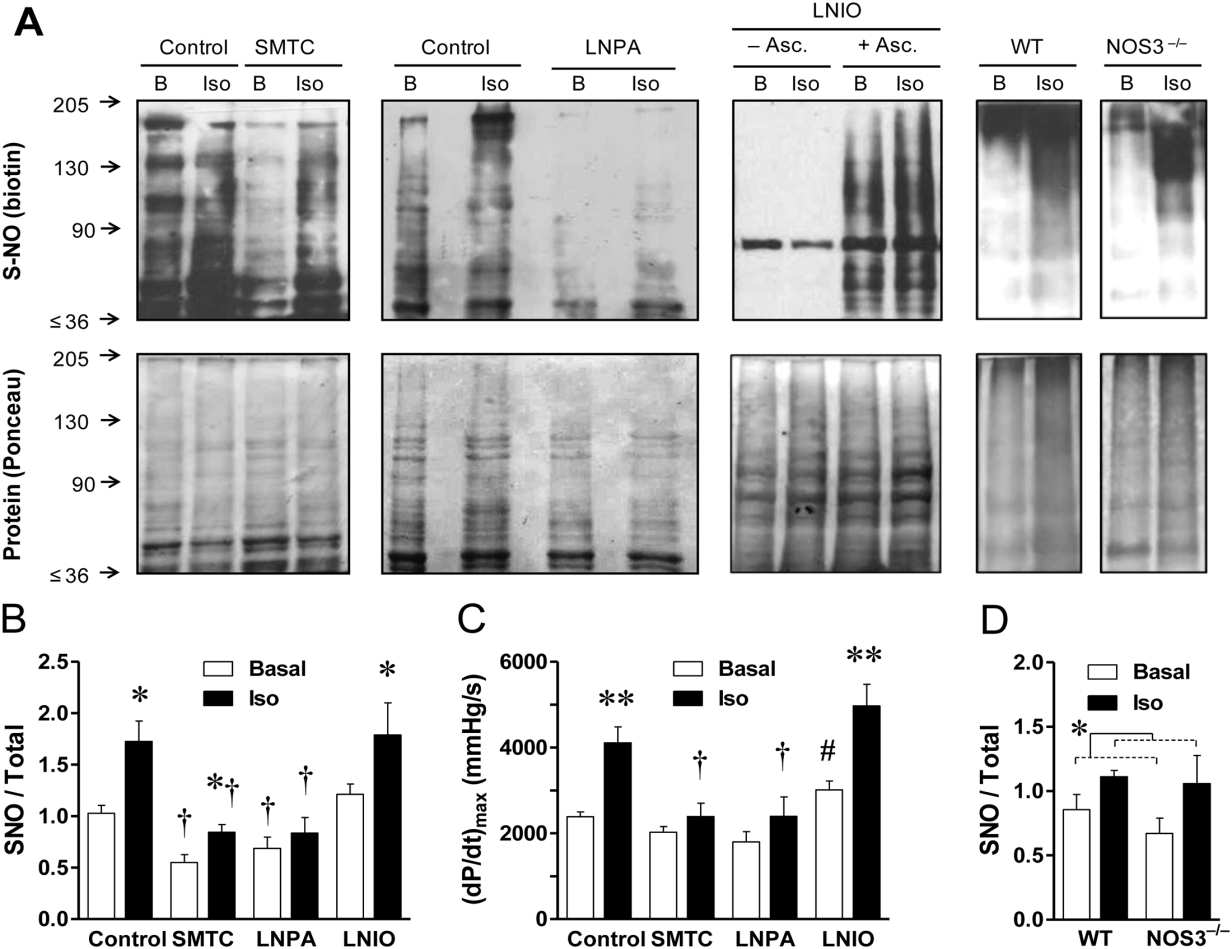
Inhibition of NOS-1 reduces basal level of protein S-nitrosylation in the heart and isoproterenol causes rapid NOS-1-dependent protein S-nitrosylation. **(A)** Representative Western blots showing protein S-nitrosylation in hearts homogenized during baseline (B), or after stimulation with isoproterenol (10nM, 3-min, Iso); in control conditions, or after 20-min treatment with NOS-1 inhibitor, L-Methyl-thio-citrulline 300nM (SMTC), or N^ω^—propyl—L—arginine 200nM (LNPA), or NOS-3 inhibitor L-N^5^-(1-Iminoethyl)ornithine 1μM (LNIO). Homogenized tissue was submitted to the biotin-switch procedure, followed by SDS-PAGE analysis. The same membranes are shown stained for biotin (top) and for total protein content (Ponceau red, bottom). A representative control for the specificity of the biotinylation reaction, is shown in the LNIO panel, where same pair of samples was incubated without ascorbate (–Asc.) or with this reducing agent (+ Asc.). The far right panels show S-nitrosylation analysis of hearts obtained from wild type (WT) and NOS-3 deficient (NOS3^–/–^) mice. **(B)** Densitometric analysis of normalized biotin/Ponceau signal for the series of rat hearts (Control n = 8, SMTC n = 8; LNPA n = 5, LNIO n = 6). All proteins included in the rectangular area (range ~35–210 KDa) were quantified. Two-way ANOVA indicated significant effect of inhibitors (p<0.005) and β-adrenergic stimulation (p<0.0005), without significant interaction. Asterisk indicates p<0.05 Iso vs. respective Basal; † indicates p<0.01 vs. corresponding Control and LNIO value. **(C)** Cardiac contractility determined in the same rat hearts immediately prior to homogenization. Two-way ANOVA indicates significant effect of inhibitors (p<0.005), isoproterenol (p<0.01), and interaction (p<0.05). ** p<0.01 Iso vs. respective Basal; † indicates p<0.01 vs. corresponding Control value; # p<0.05 LNIO vs. all other basal values. **(D)** Densitometric analysis of normalized biotin/Ponceau signal for the experimental series of WT and NOS-3^–/–^mice hearts. According to two-way ANOVA there was significant effect of Isoproterenol (*, p<0.05), but no significant effect of strain or interaction (n = 3 per group).

Next, we evaluated the effect of β-adrenergic stimulation on the degree of S-nitrosylation of cardiac proteins. For this purpose hearts were stimulated with 10nM isoproterenol (a concentration close to the EC_50_). After 3-min, hearts were rapidly removed, homogenized and submitted to biochemical analysis for S-nitrosylation of general and specific cardiac proteins. The biotin-switch assay revealed that relative to control conditions, isoproterenol induced an increase of 46±14% in the degree of S-nitrosylation, which was paralleled by a 76±14% increase in contractility (Fig 3).

To test for the role of NOS-1 in this S-nitrosylation, separate set of hearts were pre-treated with SMTC (300nM) before the challenge with isoproterenol. Interestingly, the sole inhibition of NOS-1 reduced basal S-nitrosylation in a 52±8% compared to non-treated hearts (paired experiments). Furthermore, the degree of S-nitrosylation after isoproterenol stimulation in hearts treated with SMTC was reduced by a 41±8% relative to controls, strongly suggesting a role for NOS-1 in the adrenergic-induced nitrosylation (Fig 3B). To confirm this assertion, we used another NOS-1 inhibitor, LNPA. This compound exerted a similar effect to SMTC, reducing the level of S-nitrosylation by a 25±13% in non-stimulated hearts and by 45±17% in hearts stimulated with isoproterenol. Nonetheless, in the presence of NOS-1 inhibitors, isoproterenol still caused an increment in protein S-nitrosylation, that may indicate a remnant source of NO available for nitrosylation, probably due to incomplete NOS-1 inhibition since we cannot assure that NOS-1 activity was 100% suppressed with these concentrations. Confirming the results on Fig 1, NOS-1 inhibition caused a significant reduction in the magnitude of the inotropic response to isoproterenol, amounting only to 46±12% with SMTC and 29±13% with LNPA treatment (Fig 3C).

In contrast, blockade of NOS-3 with LNIO 1μM, did not reduce protein S-nitrosylation, neither basal (+7±9%), nor after stimulation with isoproterenol (+15±20%); and consistently with our hypothesis, basal contractility was increased and the inotropic response to isoproterenol was not reduced by LNIO (85±14%, Fig 3C). Most of the biotin switch signal was suppressed by omission of ascorbate, confirming the specificity of the procedure to detect S-nitrosylation. The key participation of NOS-1 in the process was further confirmed by the observation that isoproterenol caused a similar protein S-nitrosylation increment in WT and NOS-3 deficient mice (Fig 3A and 3D). These findings extend the observations of rapid cardiac protein S-nitrosylation in response to β-adrenergic stimulation to a second species, and demonstrate lack of participation of NOS-3 in the process. Altogether these data support a direct relationship between NOS-1 mediated S-nitrosylation and contractility.

To discard a possible participation of other NO sources, we tested for the presence of inducible NOS (NOS-2) in our experimental conditions. NOS-2 was not detected in perfused hearts or livers of control rats; whereas it was detected in LPS-treated animals used as positive controls (S1 Fig).

### S-nitrosylation of calcium-handling proteins

To gain further insights into the mechanism of how S-nitrosylation contributes to cardiac contractility, we investigated S-nitrosylation of key calcium handling proteins. Hearts treated with or without SMTC were submitted to the biotin-switch assay in the baseline condition or after stimulation with isoproterenol. Biotin substituted S-nitrosylated proteins were selectively separated (streptavidin pull-down) and analyzed with specific antibodies.

**Fig 4:**
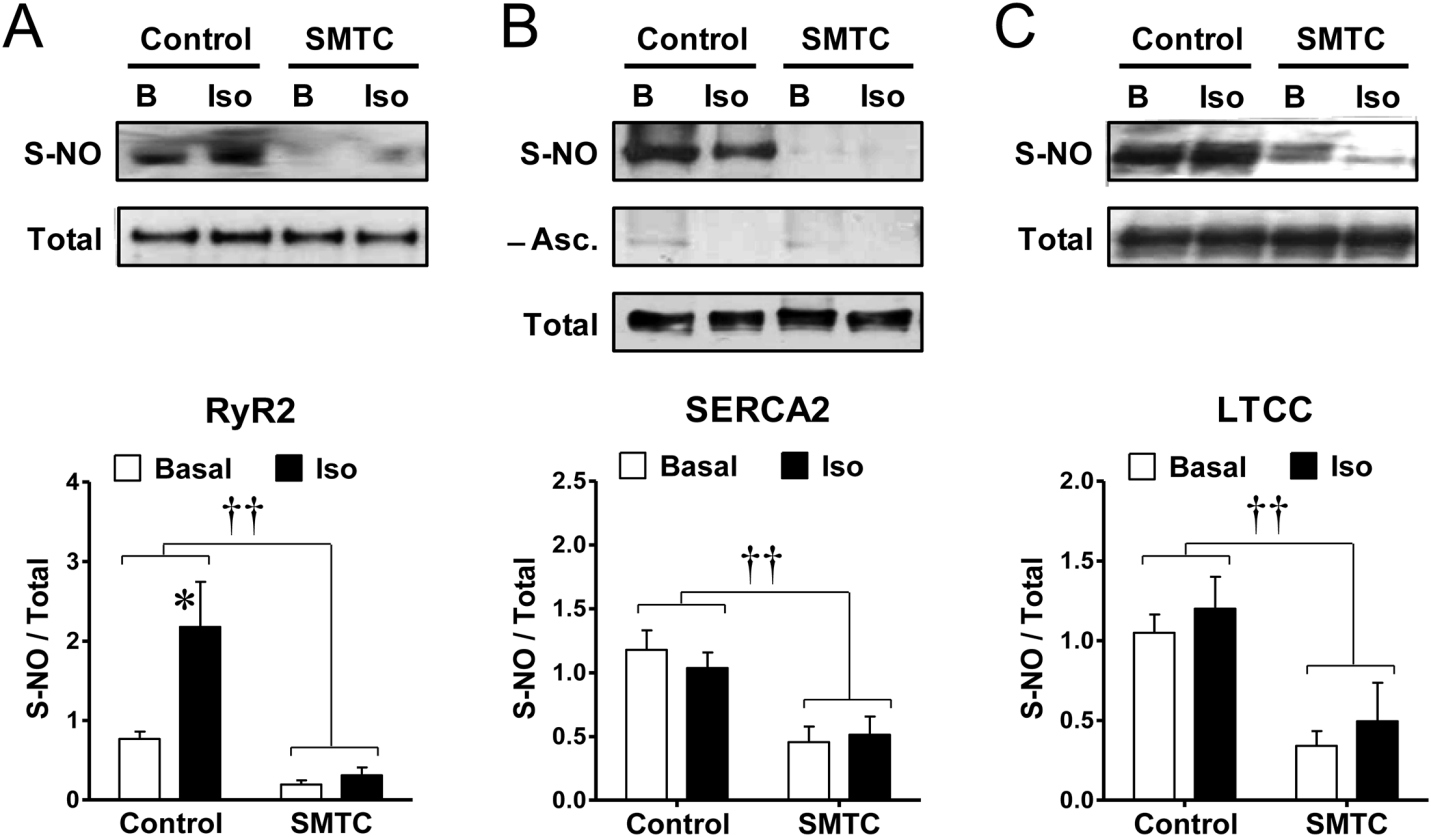
NOS-1 inhibition reduces S-nitrosylation of RyR2, SERCA2 and LTCC, and isoproterenol increases S-nitrosylation of RyR2. Hearts were homogenized prior (basal, B) or after stimulation with isoproterenol (10nM, 3-min, Iso), in the absence or presence of the NOS-1 inhibitor SMTC (300nM, 20-min). Following biotin-switch and streptavidin pull-down, proteins were probed with specific antibodies for RyR2 **(A)**, SERCA2 **(B)**, and LTCC **(C)**. **Top**: Representative Western blots for S-nitrosylated proteins (S-NO) and their matched total content in homogenate (Total). In **(B)**, the middle panel shows the signal for the same samples omitting ascorbate during the biotin-switch procedure, as negative control. **Bottom**: densitometric analyses of several similar experiments. (RyR2: Control n = 8, SMTC n = 5; SERCA2: n = 11 each, LTCC: n = 10 each). †† p<0.001 SMTC vs. control, two-way ANOVA; * p<0.05 Iso vs. respective Basal, t test.

In first place we studied RyR2, since this channel has been reported to be S-nitrosylated in response to NO donors [22, 39, 40]. The pattern of RyR2 S-nitrosylation resembled that of general cardiac proteins, but accentuated (Fig 4A). In control conditions RyR2 was endogenously S-nitrosylated and brief stimulation with isoproterenol increased this modification by 146±77%. Blocking NOS-1 with SMTC caused a 70±9% reduction in RyR2 S-nitrosylation relative to baseline, and during NOS-1 blockade isoproterenol did not increase S-nitrosylation of this protein.

Second we analyzed SERCA2, since this pump is a major regulator of cardiac contractility and it has been suggested to undergo redox modifications, including S-nitrosylation [41, 42]. The biotin switch revealed that SERCA2 was also endogenously S-nitrosylated and this nitrosylation was reduced upon treatment with SMTC by 56±13% (Fig 4B). At difference with the pattern of general proteins and RyR2, isoproterenol did not induce further SERCA2 nitrosylation neither in control nor in SMTC-treated hearts. Again the negative control without ascorbate abolished the nitrosylation signal, confirming the specificity of SERCA2 nitrosylation.

Third, we assessed S-nitrosylation of the L-type calcium channel (LTCC), as another relevant protein involved in the cardiomyocyte Ca^2+^ cycle. As seen in Fig 4C, blockade of NOS-1 activity also reduced basal S-nitrosylation of LTCC by 63±10%; however, similar to the results with SERCA2, isoproterenol did not modify LTCC S-nitrosylation.

The specific dependency on NOS-1 activity for the maintenance of S-nitrosylation of these proteins was confirmed because inhibition of NOS-3 with LNIO did not produce changes in S-nitrosylation of RyR2, SERCA2 or LTCC (S2 Fig).

### Phospholamban (PLB) phosphorylation and oligomerization

**Fig 5:**
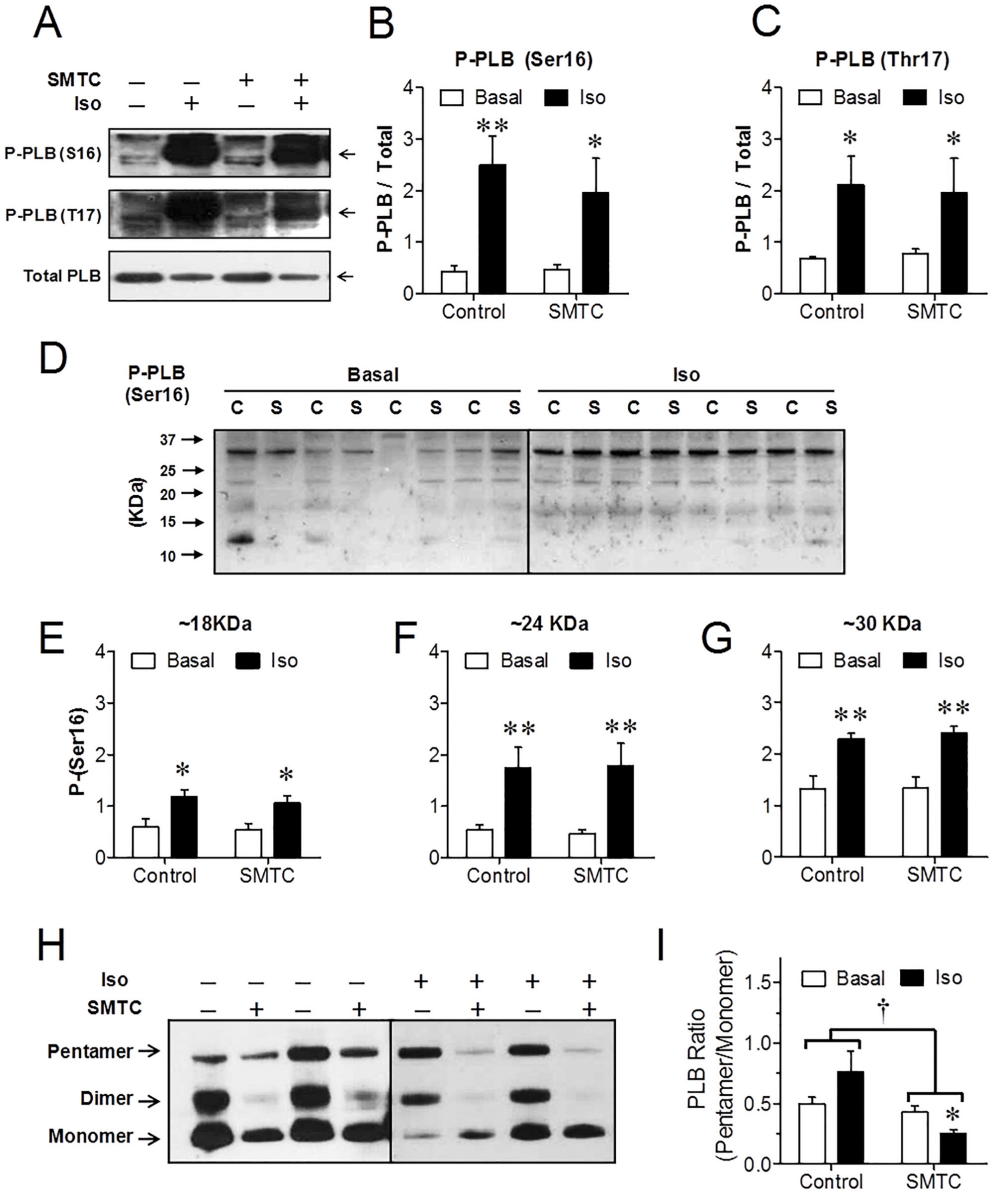
NOS-1 inhibition does not affect phosphorylation of phospholamban (PLB) in rat hearts. **(A)** Representative Western blots of PLB phosphorylated on Ser16 (top), PLB phosphorylated on Thr17 (middle) and total PLB (bottom), in hearts homogenized before (basal, B) and after stimulation with isoproterenol (10nM, 3-min, Iso), following 20-min perfusion in the absence or presence of NOS-1 inhibitor SMTC 300nM. Arrows indicate 30KDa. **(B)** Densitometric analysis of the phospho-Ser16 over total PLB content ratio (n = 6); **(C)** Densitometric analysis for phospho-Thr17 over total PLB ratio (n = 5). In both cases, there was a significant effect of β-adrenergic stimulation (p<0.001) without effect of treatment or interaction (Two-way ANOVA). * p<0.05, ** p<0.01 vs. the respective basal. **(D)** Representative broad range Western blots depicting PLB phosphorylated on Ser16 in hearts homogenized before (Basal) and after stimulation with isoproterenol (10nM, 3-min, Iso), after 20-min perfusion in control conditions (C) or with SMTC 300nM (S). Densitometric analysis of bands corresponding to PLB trimers **(E)**, tetramers **(F)** and hexamers **(G)** indicate significant effect of Iso, but not of SMTC, nor interaction (Two-way ANOVA; * p<0.05, ** p<0.01 vs. the respective basal, n = 6–8 hearts per group). **(H)** Representative blots of PLB in hearts treated with or without isoproterenol, in the presence or absence of SMTC. **(G)** Analysis of pentamer/monomer intensity ratio from 7–9 similar experiments per group. Two-way ANOVA indicated significant effect of NOS-1 inhibition (†, p<0.01), and interaction between treatment and β-adrenergic stimulation (p<0.05). * p<0.05 vs. Control, and vs. Control-Iso (Newman-Keuls post hoc test).

Phosphorylation of PLB on serine 16, dependent on protein kinase A activation [1, 42], and on threonine 17, dependent on Ca^2+^/calmodulin kinase II [43, 44], is a primary mechanism by which adrenergic stimulation increases SERCA activity, thereby increasing Ca^2+^ reuptake into the SR. It has been suggested that NOS-1 may exert inotropic effects through modulation of PLB phosphorylation [18, 45]. Therefore, we investigated whether NOS-1 inhibition, together with impairing S-nitrosylation may have interfered with PLB phosphorylation induced by β-adrenergic stimulation, affecting contractility by that pathway. Treatment with SMTC neither caused changes in the basal level of PLB phosphorylation nor altered the increase in phosphorylation upon adrenergic stimulation on both Ser16 and Thr17 (Fig 5A–5C). Since basal PLB phosphorylation has been reported to be reduced in NOS-1 KO mice or after NOS-1 inhibition [18, 45], we focused on analysing the level of baseline P-Ser 16 PLB in control and SMTC treated hearts. Systematically and consistently we did not find differences in PLB phosphorylation, in any multimer arrangement in perfused rat hearts (Fig 5D–5G). Nevertheless, since it was recently described that S-nitrosylation of PLB is necessary for PLB pentamerization (and hence, for SERCA disinhibition) upon β-adrenergic stimulation [8], we analysed the distribution of PLB oligomeres. Overall, SMTC reduced significantly PLB pentamerization, evaluated as the pentamer/monomer intensity ratio (Fig 5H and 5I). While at baseline there was no difference, the effect of NOS-1 inhibition on pentamerization was evident in isoproterenol treated hearts.

### NOS-1 phosphorylation

**Fig 6:**
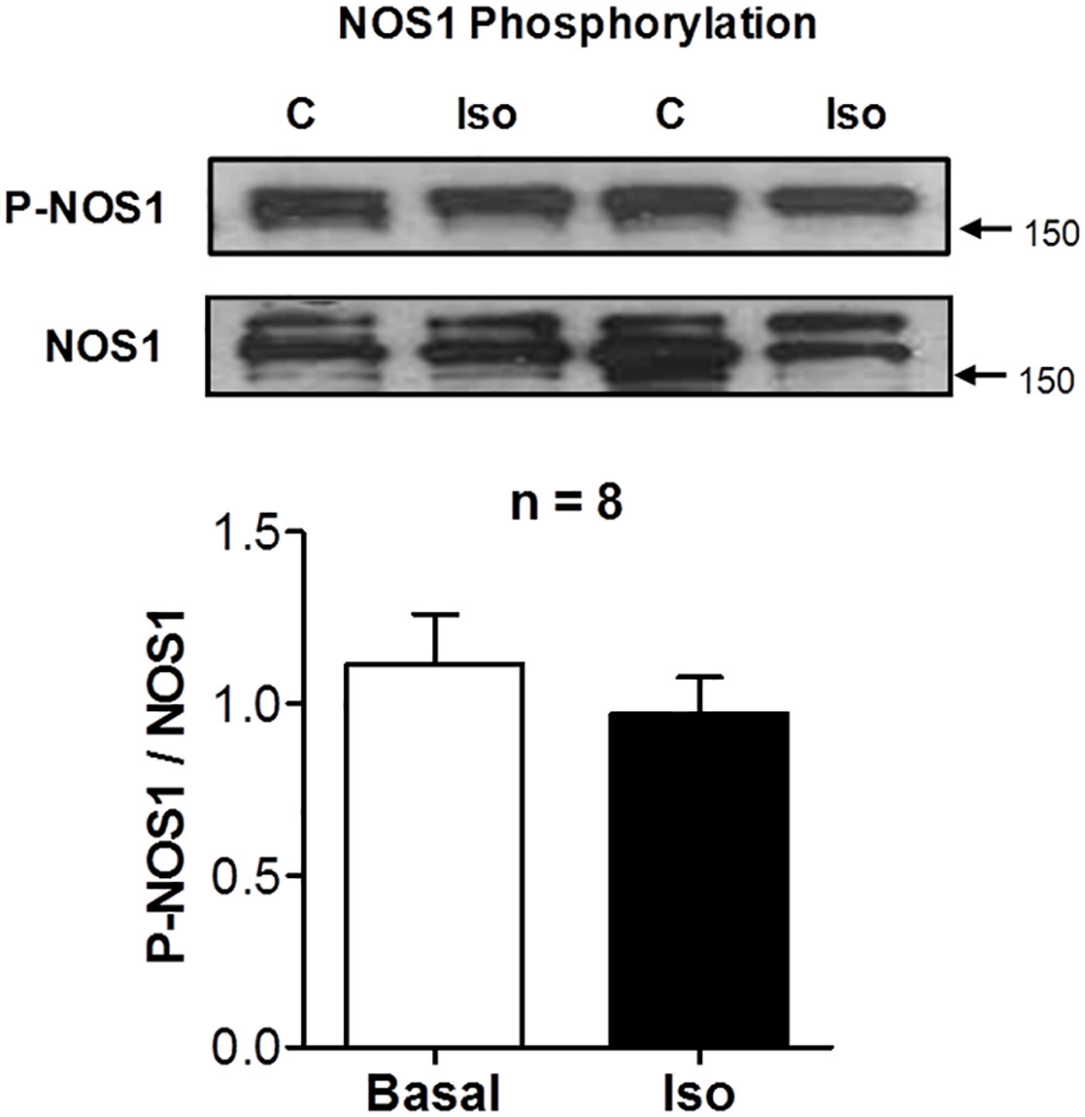
Isoproterenol does not modify NOS-1 phosphorylation. Representative Western blot of NOS-1 phosphorylated at Ser1412, and corresponding total NOS-1 content in hearts homogenized before (control, C) and after stimulation with isoproterenol (10nM, 3-min, Iso). The graph shows densitometric analysis of the phospho-NOS-1 over total NOS-1 content ratio (n = 8). No differences were detected.

The current results clearly show that increased protein S-nitrosylation, including RyR2, following adrenergic stimulation required NOS-1 activity; thus we investigated for possible links between β-adrenergic signaling and NOS-1 activity leading to S-nitrosylation. It has been reported that β-adrenergic signaling causes NO production in rabbit cardiac myocytes through Akt phosphorylation of NOS-1 on serine S1416, activating this enzyme [21]. Also rat NOS-1 can be phosphorylated at serine S1412 by protein kinase A, resulting in an increased NOS activity [46]. Thus, we addressed the question as to whether in the conditions used in our experiments, β-adrenergic stimulation caused NOS-1 phosphorylation at serine S1412. However, in two independent series of experiments, there was no difference in the level of NOS-1 phosphorylation prior and after challenging the hearts with 10nM isoproterenol, as shown in Fig 6. Therefore, in absence of a distinct test to measure a rapid increase in NOS-1 activity that may account for rapid S-nitrosylation; our results favor an alternative hypothesis, which is that the basal level of enzyme activation is obligatory but not sufficient for eliciting S-nitrosylation, and a secondary pathway, for instance increases in oxidative stress in the vicinity of target proteins, actually triggers the increment in this modification after β-adrenergic stimulation.

### Effect of Reactive Oxygen Species (ROS) on S-nitrosylation and contractility

**Fig 7:**
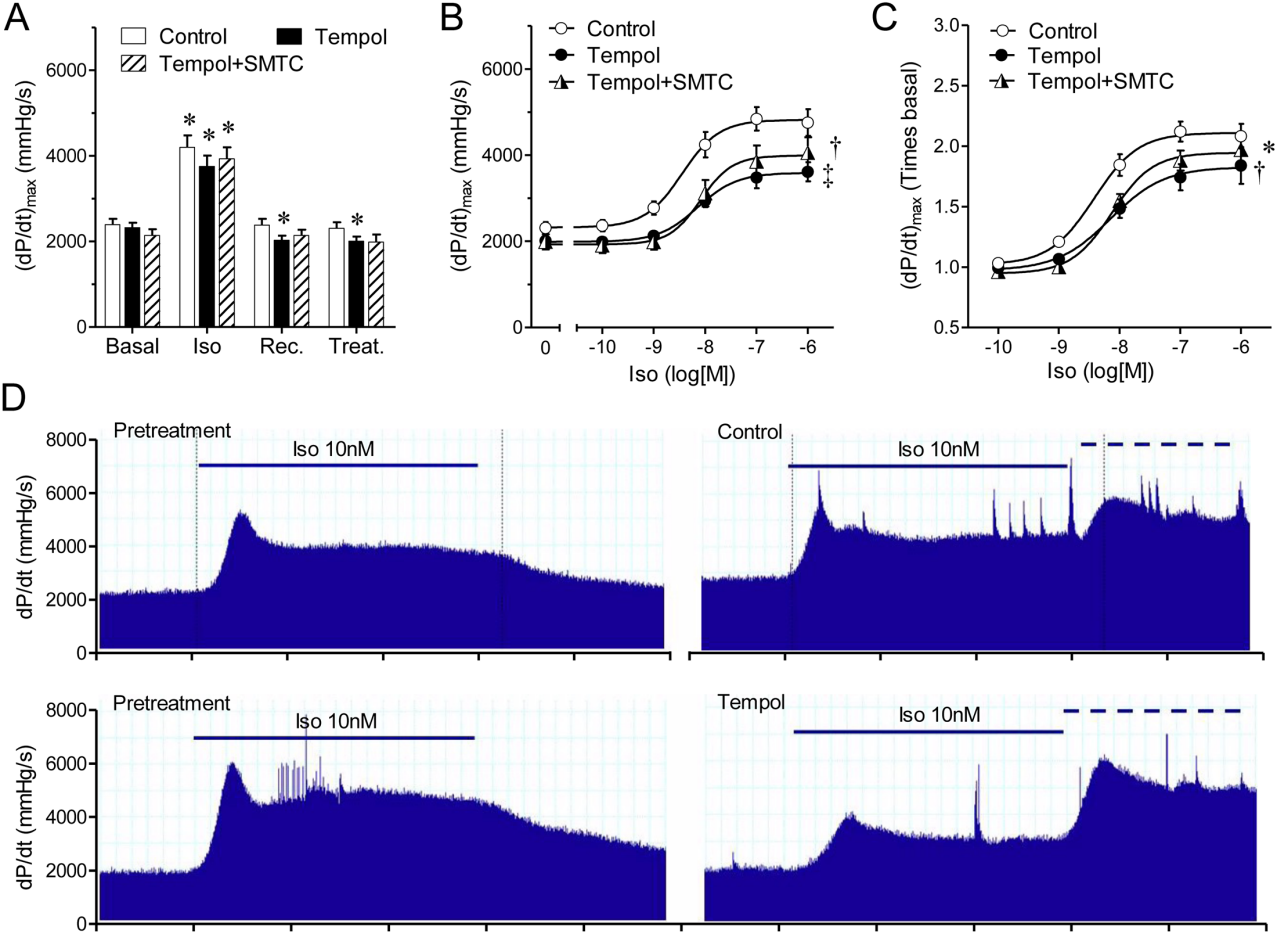
Inhibition of the nitrosylation reaction with tempol, a radical scavenger, reduces the inotropic response to β-adrenergic stimulation. **(A)** Contractility (dP/dt_max_) determined consecutively under basal conditions (Basal), isoproterenol stimulation (Iso); recovery (Rec.), and treatment (Treat.) with vehicle (Control, n = 12), tempol 100μM (Tempol, n = 9), or tempol 100μM plus NOS-1 inhibitor SMTC 300nM (Tempol+SMTC, n = 6), details as in Fig 1. Asterisk indicates p<0.05 vs. Basal (paired t test, with Dunnett’s modification). **(B)** Concentration-response curves to isoproterenol alone (Control), in the presence of tempol, and tempol plus SMTC. **(C)** Same data as in panel B, expressed as relative values, corrected by the corresponding contractility values after treatment with inhibitor or inhibitors. * p<0.05, † p<0.01, ‡ p<0.001 vs. Control curve (Best fit ANOVA, the difference resided only in the top value; bottom and EC_50_ parameters were shared). **(D)** Representative records showing contractility changes (dP/dt) elicited by the application of Iso10nM (solid line) before and after treatment (control or tempol). The dashed line indicates application of 100nM Iso as part of the dose-response curve.

Thus, to evaluate the role of reactive oxygen species and S-nitrosylation in the contractile response induced by isoproterenol, we treated hearts with the membrane-permeable radical scavenger tempol, which inhibits the S-nitrosylation reaction. As for the case of NOS-1 inhibitors, treatment with tempol reduced basal contractility (Fig 7A), developed pressure and lusitropy, and increased perfusion pressure to a similar extent as SMTC and LNPA (Table 1). Pre-treating hearts with tempol (100μM 20-min), reduced the response to isoproterenol, shifting the concentration response curve downward, without affecting the EC_50_, similarly to the NOS-1 inhibitors (Fig 7B). Co-application of tempol and SMTC did not produce a further reduction in the inotropic response to isoproterenol, suggesting that NOS-1 inhibition and blocking of S-nitrosylation partake in the same signaling pathway. Despite the reduced basal contractility after treatment with tempol, or tempol plus SMTC, the inotropic response elicited by isoproterenol in these conditions was significantly smaller than in control when we analyzed the increment in relative units corrected by the respective baseline (Fig 7C).

**Fig 8:**
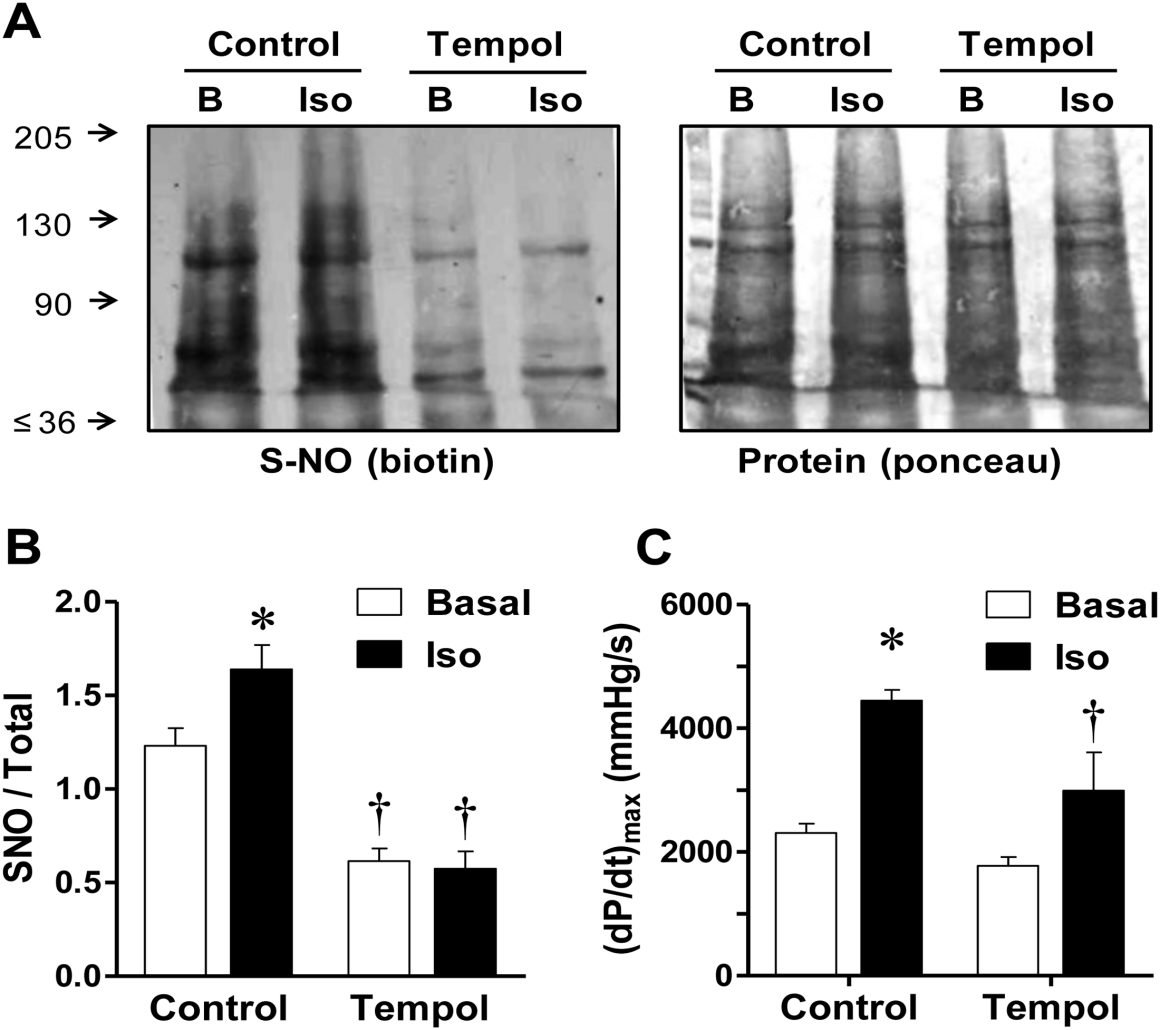
Tempol reduces protein S-nitrosylation and cardiac contractility. **(A)** Representative Western blots showing protein S-nitrosylation in hearts homogenized prior (basal, B), or after stimulation with isoproterenol (10nM, 3-min, Iso), in control conditions or after 20-min treatment with tempol (100μM). Homogenized tissue was submitted to the biotin-switch assay and SDS-PAGE. The same membrane is shown stained for biotin (left) and for total protein (right), as detailed in the legend to Fig 3. **(B)** Densitometric analysis of the normalized biotin/total protein signal for 8 similar experiments. Two-way ANOVA indicated significant effect of tempol (p<0.0001) and interaction (p<0.05). **(C)** Cardiac contractility determined in the same hearts immediately previous to homogenization. Two-way ANOVA indicated significant effect of tempol (p<0.01), and isoproterenol (p<0.001) without significant interaction. * p<0.05, ** p<0.01 vs. respective Basal; †, p<0.01 vs. corresponding Control value.

Thereafter, we investigated whether tempol affected S-nitrosylation in this system. Treatment with tempol decreased basal S-nitrosylation by 60±6% and reduced the isoproterenol-induced S-nitrosylation by a 75±5%, and again, these reductions in nitrosylation were mirrored by a weak contractile response to the β-adrenergic agonist (only 29±13% increment, Fig 8), reinforcing the concept that this posttranslational modification plays an important role both on basal contractility and on the contractile reserve triggered by adrenergic stimulation. The finding that the effects of NOS-1 inhibitors on S-nitrosylation and contractility were mimicked by tempol strongly suggests that S-nitrosylation in addition to NOS-1 activity, requires a source of superoxide [47].

**Fig 9:**
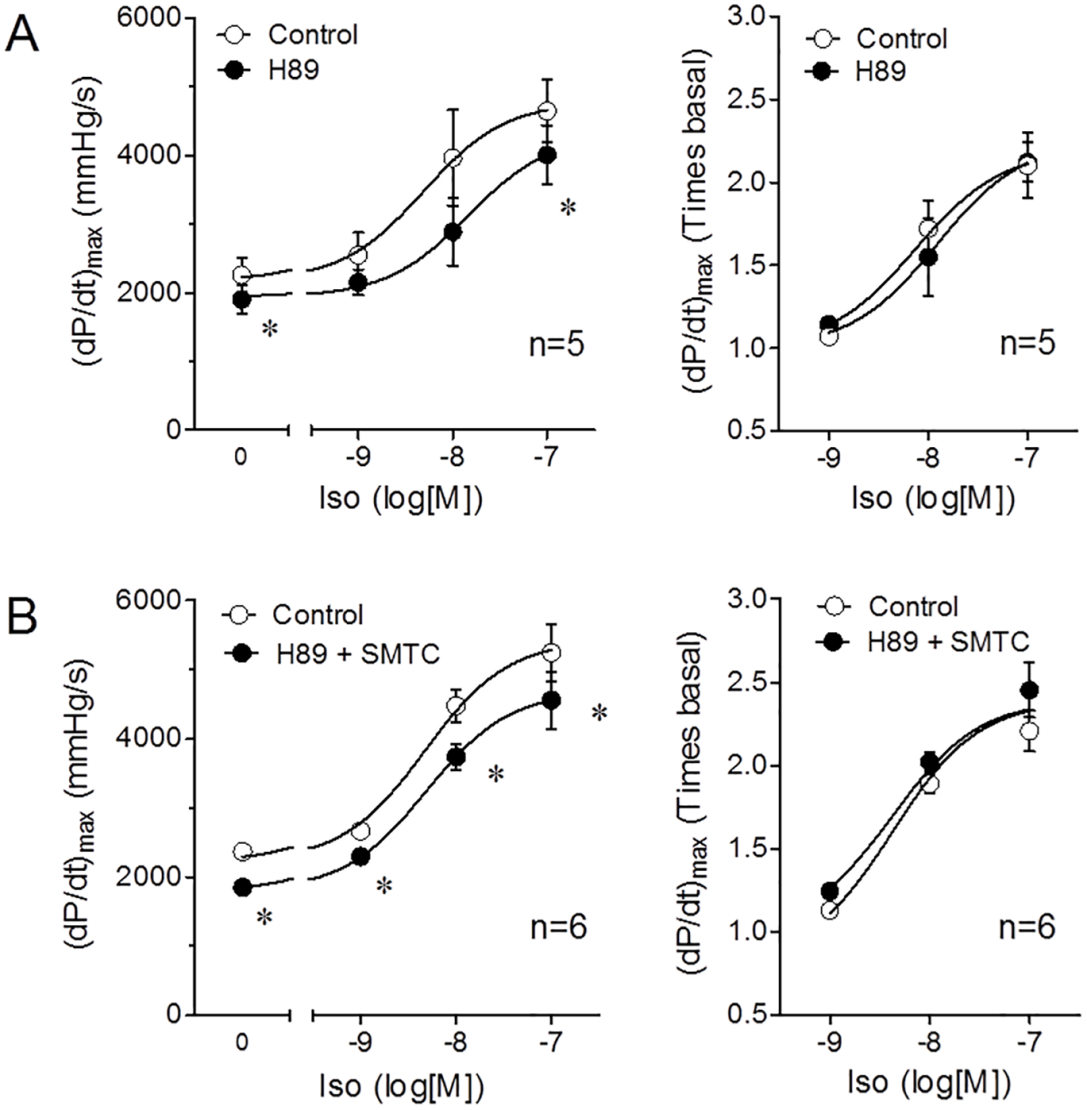
PKA blockade prevents contractility reduction induced by NOS-1 inhibition. Accumulative concentration-response curves of heart contractility (dP/dt)max to isoproterenol prior (Control) or after adding PKA inhibitor H89 0.5μM **(A)**; or H89 0.5μM plus NOS-1 inhibitor SMTC 300nM **(B)**. The same data are expressed in absolute values **(Left)** or as relative values, corrected by the corresponding contractility values after treatment with inhibitors **(Right)**. (* p<0.01 vs. Control, paired t test). Best fit parameters could not be calculated because 1μM isoproterenol was not tested (lack of saturation).

Therefore, ROS production is a likely link between β-adrenergic stimulation and NOS-1 dependent S-nitrosylation. Because in isolated mouse cardiomyocytes it was shown that isoproterenol increases mitochondrial ROS production via the cAMP-PKA pathway [48] we analyzed the effect of NOS-1 inhibition during PKA inhibition using a pharmacological inhibitor of PKA, H89. As shown in Fig 9, PKA inhibition reduced basal contractility (-16±4%) and displaced the isoproterenol concentration-response curve downstream; but the fractional increment in contractility induced by isoproterenol was not different from control. Surprisingly, in the presence of H89, SMTC did not induce a further reduction in either baseline or isoproterenol induced contractility as compared with H89 alone (Fig 9B). These results, along with the data of ROS inhibition with tempol, suggest that PKA and ROS converge to NOS-1 dependent S-nitrosylation signaling.

In addition, to test for a role for the NO-guanylate cyclase-PKG pathway in the modulation of the response to isoproterenol by NOS-1 activity, we applied SMTC to hearts treated with the pharmacological inhibitor of guanylate cyclase, ODQ 10μM, alone (to suppress PKG activation) or ODQ 10μM plus the cGMP soluble analog 8Br-cGMP 1μM (to maintain PKG activity constant). We choose 100nM isoproterenol since the effect of NOS1 inhibition was evidenced at the maximal response. Under both guanylate cyclase inhibition and PKG clamping, NOS-1 inhibition caused a 15–25% reduction in basal contractility and in the response to Iso (Table 2). These data suggest that NOS-1 modulation of isoproterenol induced contractility is independent of PKG signaling.

**Table 2:** NOS-1 inhibition reduces cardiac contractility independently of guanylate cyclase activity.

	**Pre-Treatment**	**ODQ + SMTC**	
**Basal**	2,413 ± 153	2,130 ± 125 †	n = 5
**Iso**	5,063 ± 153 *	4,458 ± 178 * †	n = 5
	**Pre-Treatment**	**ODQ + cGMP + SMTC**	
**Basal**	2,623 ± 138	2,090 ± 190 †	n = 5
**Iso**	4,607 ± 581 *	3,537 ± 482 * †	n = 5

Table 2: Values of maximal rate of pressure development ((dP/dt)_max_), in control conditions (Pre-Treatment) either at the end of a 20-min equilibration period (Basal) or after a 3-min stimulus with 100nM Isoproterenol (Iso). After 10-min for washout, hearts were perfused 20-min with a combination of inhibitors, followed by a second challenge with 100nM Isoproterenol in the presence of the inhibitors. In the first series, we inhibited guanylate cyclase with ODQ 10μM and NOS-1 with SMTC 300nM; in the second series, in order to clamp PKG activation, we applied ODQ 10μM, plus 8Br-cGMP 1μM, and inhibited NOS-1 with SMTC 300nM.

* p<0.001 vs. respective basal

^†^ p<0.05 vs. pre-treatment

## Discussion

The main findings of the present investigation are: first, that NOS-1 activity maintains a basal level of S-nitrosylation of Ca^2+^-handling proteins RyR2, SERCA2 and LTCC, as well as general cardiac proteins, and these post-translational modifications are associated with resting contractility and lusitropy, which are one-third higher than those observed when NOS-1 activity is suppressed; and second, that β-adrenergic stimulation of the heart induces rapid S-nitrosylation of proteins, notably of RyR2. This modification depends on NOS-1 activity and the redox environment (ROS production), and is required for full development of β-adrenergic stimulated contractility.

To discern between endogenous effects of NOS-1 and NOS-3 isoforms in the heart, we used inhibitors (SMTC, LNPA or LNIO) at concentrations that cause major inhibition of the specific isoform we were studying, without significantly affecting other isoforms. This criterion was based on reported IC_50_ values, SMTC IC_50_ is 300 nM for NOS-1 (brain slices) or 47 nM (brain cytosol) vs. 5.4 μM for NOS-3 (aortic rings) [23]. Likewise, IC_50_ of LNPA is 57 nM for NOS-1 and 8.5 μM for NOS-3 [25]. Therefore, the chosen concentrations of 300nM SMTC and 200nM LNPA are clear-cut as NOS-1 selective inhibitors, without affecting NOS-3. In this regard, SMTC has been utilized to specifically inhibit NOS-1 in hearts of guinea pig [49] and rats [50]. In the case of LNIO, reported IC_50_ are 0.5 μM for NOS-3 [51]; and 3.9 μM for NOS-1 [27]. Thus, at 1 μM, LNIO is not supposed to significantly inhibit NOS-1.

Using these NOS-1 and NOS-3 inhibitors we obtained cardiac contractility changes in agreement with the observations by Barouch and colleagues, of impaired cardiac reserve in response to isoproterenol in mice genetically deficient in NOS-1, and increased inotropic response in NOS-3 deficient mice [13]. In this regard, the effects of acute inhibition of NOS-1 and NOS-3 provide complementary evidence for the involvement of these enzymes in cardiac inotropism in an experimental model devoid of interference from changes in protein expression that may hinder interpretation in models based in chronic genetic deletions. For instance, controversy persists on the functional relevance of cardiac NOS isoforms after elegant studies involving NOS-1 and NOS-3 null mice, including appropriate genetic background [52]. Possible interferences from NOS-2 in our system were ruled out due the minimal/undetectable presence of this isoform in control hearts (S1 Fig).

S-nitrosylation has arisen as an important mechanism for regulation of the cardiac function and disruption of this signaling may have pathophysiological consequences, such heart failure and arrhythmias [17, 31, 53]. Previously, we showed that S-nitrosylation of RyR2 and other proteins induced by an NO donor (SNAP, in low concentration) was associated with an increase in inotropism, in a way independent of the cGMP-PKG pathway and of changes in PLB phosphorylation [22]. Likewise, addition of SNAP mimicked the effect of NOS-1 to activate CaMKII, resulting in increased SR Ca^2+^ leak [21]. In this work, we present evidence for short-term physiological regulation of cardiac contractility through NOS-1 mediated S-nitrosylation of RyR2, SERCA, LTCC and other cardiac proteins. Inhibition of NOS-1 reduced the magnitude of the inotropic response elicited by isoproterenol, to a similar extent that reduced adrenergic-stimulated protein S-nitrosylation, and suppressed S-nitrosylation of RyR2. Indeed, changes in S-nitrosylation of RyR2 closely followed the changes in cardiac contractility, which is consistent with the concept that this modification increases open probability of this channel [39, 40] and enhances contractility. On the other hand, it has been reported that hypo-nitrosylation and increased oxidation of RyR2 (that further increase RyR2 open probability) in pathological conditions that impose chronic oxidative stress may lead to diastolic Ca^2+^ leak and arrhythmia [17, 41]. Thus, we may consider that in minute-to-minute physiological regulation of cardiac contractility, RyR2 S-nitrosylation fluctuates between a resting NOS-1-dependent baseline level and a β-adrenergic stimulated level, which is also dependent on NOS-1 activity, and more importantly, this rapid increase in RyR2 S-nitrosylation is key to attain maximal cardiac reserve.

NOS-1 inhibition reduced basal SERCA2 S-nitrosylation by 60%, and this condition was maintained in isoproterenol stimulated hearts (Fig 4B). Therefore SERCA2 hypo S-nitrosylation correlates with the observed reduction in calcium uptake at different isoproterenol concentrations. This result is in line with reports of decreased cardiac relaxation associated to a reduced SERCA2 S-nitrosylation in hearts of capsaicin-denervated rats [54].

Diminished S-nitrosylation of LTCC may reduce cytosolic Ca^2+^ influx during the cardiac cycle [55], impairing the capacity of the cardiomyocyte to fully respond to β-adrenergic stimulation. In an earlier study β-adrenergic stimulation has been found to cause S-nitrosylation of LTCC, associated with a reduced calcium current [56], but instead, we observed no difference, similar to a more recent report [57]. Possible explanation for this discrepancy may reside in slight technical differences, for instance, Sun et al did not pulled down S-nitrosylated proteins for quantification; or may depend on differences in the cardiomyocyte redox conditions between control animals ([57], this report), and animals submitted to chronic [50, 54] or acute [56] manoeuvres affecting oxidative stress.

We show that the increment in S-nitrosylation of RyR2 and other proteins caused by β-adrenergic stimulation occurs rapidly (3-min), and we deduce that this modification is reversible, since all the hearts studied were initially challenged with isoproterenol, followed by a 30-min lapse to the moment chosen to assess basal S-nitrosylation. In addition, S-nitrosylation was largely reduced after 20-min NOS-1 inhibition, indicating active turnover of this modification. The exact time-course of de-nitrosylation remains unknown, and may vary from protein to protein.

The mechanisms that govern this rapid and reversible NOS-1 dependent S-nitrosylation remain to be determined. The degree of S-nitrosylation in the heart is regulated by the S-nitrosoglutathione reductase system (GSNOR) [57]. Similar to our results, Beigi et al. showed baseline S-nitrosylation of RyR2, SERCA and LTCC; and found that a 10-min challenge with 2.5nM isoproterenol did not alter SERCA and LTCC nitrosylation. However, opposite to our results, they reported a decrease in RyR2 nitrosylation, which may indicate that sampling time may be critical; reinforcing the idea that S-nitrosylation for RyR2 is reversible and enzymatically-controlled. Recently, Irie et al. reported that enhanced de-nitrosylation of calcium handling proteins in cardiomyocytes from mice over expressing GSNOR, or during NOS inhibition resulted in reduced Ca^2+^ transients and sarcomere shortening in response to isoproterenol [8]. They reported S-nitrosylation in response to β-adrenergic stimulation of PLB, troponin C and the sodium calcium exchanger (NCX), detected using the biotin switch method; however, at difference with our results, Irie et al. did not find changes in S-NO of RyR2 with this method; nevertheless, when using a phenyl mercury resin-assisted capture of S-nitrosylated cysteines they found increases in S-nitrosylation of RyR2, SERCA and LTCC, which were abrogated by GSNOR over-expression [8]. These discrepancies emphasize the difficulties in assessing S-NO dynamics in complex systems, such as the beating heart. Additionally, GTP cyclohydrolase-1, an enzyme involved in the biosynthesis of tetrahydrobiopterin (a co-factor of NOS) has been described involved in the regulation of NOS-1 activity and RyR2 S-nitrosylation [58].

There are several reports indicating that NOS-1 deletion or inhibition causes a reduction in PLB basal phosphorylation in mice [18, 45], modification that has been attributed to contribute to lower inotropism; however, the same result has not been found in the rat [50, 59], similar to our results. We found that NOS-1 dependent S-nitrosylation increases contractility by a mechanism not involving altered PLB phosphorylation. Interestingly, Irie et al, using a genetic model of hypo-nitrosylation, described that β-adrenergic stimulation caused NOS dependent S-nitrosylation of PLB, and this modification was determinant for pentamerization of this regulatory protein and SERCA activation, without changes in PLB phosphorylation in response to isoproterenol [8]. Although we did not study PLB S-nitrosylation, in agreement with their observation, we found that NOS-1 inhibition also reduced PLB pentamerization in response to isoproterenol, consistent with NOS-1 induced facilitation of contractility. The possibility that changes in SERCA2 S-nitrosylation affect the interaction between this pump and PLB remains an open question.

Desensitization is a mechanism by which β-adrenoreceptors (and other G-protein-coupled receptors) regulate their activity. G-protein-coupled receptor kinase-2 (GIRK2) and β-arrestin are S-nitrosylated upon β-adrenergic stimulation [60]. Although this is an important mechanism by which S-nitrosylation prevents attenuation of the β-adrenergic stimulation, desensitization is not a mechanism involved in the loss of response to isoproterenol in our protocols. First, control and treated hearts were submitted to the same sequence of two bouts of stimulation, second, stimulation with the agonist lasted only 3-min, precluding β-adrenergic receptor desensitization. In addition, the levels of phospholamban phosphorylation on Ser-16, which is activated by the cAMP-PKA cascade [1], were not altered by loss of nitrosylation indicating that the coupling between agonist stimulation and PLB phosphorylation was not affected. Additionally, S-nitrosylation of β-arrestin and GIRK2 is mediated by NOS-3 [60, 61], and our experiments show that loss of contractility is the result of NOS-1 inhibition.

Our results demonstrate that NOS-1 activity is absolutely required for increased S-nitrosylation of RyR2 after β-adrenergic stimulation, but this process does not necessarily involve activation of the enzyme. In fact, we could not detect changes in NOS-1 phosphorylation at S1412 prior and after isoproterenol. Therefore, we favor the notion that S-nitrosylation is set off by a secondary pathway, likely changes in the redox environment and/or oxidative stress in the vicinity of target proteins, provided that NOS-1 is active and properly located. Precise location of NOS-1 has been reported to exert key function in normal and pathologic myocardium [9, 62]. Mitochondrial oxygen radical signalling has been demonstrated in cardiomyocytes contributing to the inotropic response after β-adrenergic stimulation [48], and/or causing Ca^2+^ waves [48, 63], and ROS are also produced in the SR via NAPDPH activity in response to physiological stretch [64]. It is likely that a similar mechanism may explain why only RyR2 but not SERCA2, another protein located at the sarcoplasmic reticulum is S-nitrosylated after β-adrenergic stimulation. It is also interesting that LTCC, being closely located to RyR2 in the diad (about 20 nm apart) is not nitrosylated upon β-adrenergic stimulation. Although we do not have a clear response for this phenomenon, several hypotheses arise. For instance, it has been shown RyR2 is located (on average) at 200 nm from NOS1, while the distance between RyR2 and eNOS (located in caveolae in the plasma membrane) is 370 nm. In other words, this would be approximately the distance between LTCC and the source of NO, in this case NOS1 (nNOS) [65]. Another possibility is that the β-adrenergic induced S-nitrosylation is not governed by diffusional principles, but by transnitrosylation [66]. This would imply an intermediary source of SNO after NOS1. Not surprisingly, it has been reported that NOS1 interacts with GSNOR in the sarcoplasmic reticulum [57].

The importance of changes in the redox environment for a full contractility response to isoproterenol was further confirmed by the effects of Tempol, which is a SOD mimetic able to inhibit the S-nitrosylation reaction when this involves radical intermediates such as thyil radicals [47]. Tempol reduced basal and stimulated contractility and S-nitrosylation to a similar extent as NOS-1 inhibitors, and both agents did not show synergism. Consistent with this idea, it was reported that cAMP-PKA dependent mitochondrial ROS signaling contributed by approximately 30% of maximal cell shortening and [Ca^2+^]_i_ transient, in response to isoproterenol, as determined by antioxidant treatment (55), in line with our results using NOS-1 inhibition or tempol. This prompted us to test whether PKA signaling may partake in the S-nitrosylation process. The finding that PKA inhibition abolished SMTC reduction of inotropism supports the concept that β-adrenergic stimulation causes increased ROS production, which in the presence of active NOS-1 causes RyR2 and other protein S-nitrosylation and increased [Ca^2+^]_i_ release. Interestingly, in the presence of tempol there was no remnant increment in protein S-nitrosylation after β-adrenergic stimulus, as was seen with SMTC and LNPA, reinforcing the idea of a key role for oxidative stress as the driven signal for the posttranslational modification.

Consistent with the observation that NOS-1 derived S-nitrosylation is important for the adrenergic response of the heart, recent reports suggest that NOS-1 is critical for the cardiac adaptation to exercise. Indeed, Roof et al described that exercise increases contractility and the amplitude of calcium transients in a way that is NOS-1 dependent [67, 68]. In addition, recently it has been shown that NOS-1 is absolutely necessary for the cardiac response to stretching [65]. This response consisted of increased RyR2 sensitivity that increased Ca^2+^ sparks and transients. It is tempting to speculate that this increased RyR2 sensitivity is a NOS1-dependent S-nitrosylation.

We conclude that full activation of the cardiac reserve, a central mechanism in the context of the “fight or flight” response of the heart [69] requires rapid and reversible NOS-1 dependent S-nitrosylation. On the other hand, it would be no surprising that overactivation of this same mechanism may generate nitrosative/oxidative stress, with deleterious effects, as it is well established for chronic adrenergic stimulation.

## Supporting Information

S1 Fig Hearts of normal rats do not present significant levels of inducible NOS (NOS-2). The presence of NOS-2 was assessed in heart and liver homogenates of normal rats, or rats injected with LPS (3mg/Kg i.v.) and analyzed after 2.5 hr., as positive controls. As shown in the representative Western blots, a strong (liver) or weak (heart) specific NOS-2 band was detected only in LPS-treated rats.
(TIF)

S2 Fig NOS-3 inhibition does not modify basal S-nitrosylation of RyR2, SERCA2 and LTCC, and does not alter isoproterenol-induced increase in S-nitrosylation of RyR2. Hearts were homogenized prior (basal, B) or after stimulation with isoproterenol (10nM, 3-min, Iso), in the absence (control) or after 20 min perfusion with NOS-1 inhibitor SMTC (300nM) or NOS-3 inhibitor LNIO (1μM). Proteins were probed with specific antibodies for RyR2, SERCA2 and LTCC after biotin-switch and streptavidin pull-down. **(A)** Representative Western blots for S-nitrosylated proteins (S-NO) and their matched total content in homogenate (Total). **(B)** Densitometric analyses of S-NO/total signal intensity ratio in LNIO-treated hearts (n = 4) Signal intensity was normalized to the value of untreated, unstimulated hearts in the same blots (n = 3, dashed line). * p<0.05 Iso vs. respective Basal, t test.
(TIF)
