# Supplementary figures and images for: Nitric Oxide Synthase 1 Modulates Basal and β-Adrenergic-Stimulated Contractility by Rapid and Reversible Redox-Dependent S-Nitrosylation of the Heart
Source: PLoS One. 2016 Aug 16;11(8):e0160813. doi: 10.1371/journal.pone.0160813 (PMC4986959; doi:10.1371/journal.pone.0160813)

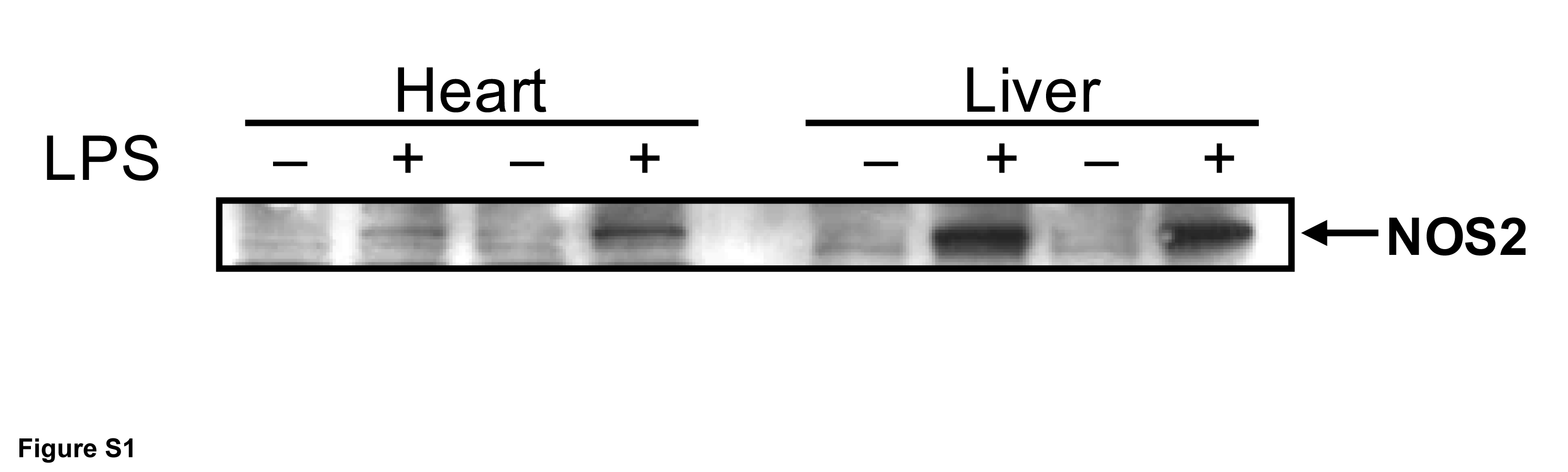

Supplement: S1 Fig — The presence of NOS-2 was assessed in heart and liver homogenates of normal rats, or rats injected with LPS (3mg/Kg i.v.) and analyzed after 2.5 hr., as positive controls. As shown in the representative Western blots, a strong (liver) or weak (heart) specific NOS-2 band was detected only in LPS-treated rats. (TIF) [file pone.0160813.s001.tif]

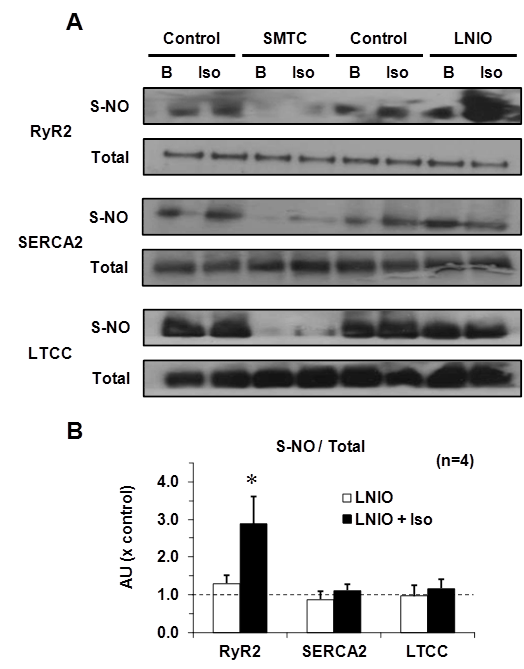

Supplement: S2 Fig — Hearts were homogenized prior (basal, B) or after stimulation with isoproterenol (10nM, 3-min, Iso), in the absence (control) or after 20 min perfusion with NOS-1 inhibitor SMTC (300nM) or NOS-3 inhibitor LNIO (1μM). Proteins were probed with specific antibodies for RyR2, SERCA2 and LTCC after biotin-switch and streptavidin pull-down. (A) Representative Western blots for S-nitrosylated proteins (S-NO) and their matched total content in homogenate (Total). (B) Densitometric analyses of S-NO/total signal intensity ratio in LNIO-treated hearts (n = 4) Signal intensity was normalized to the value of untreated, unstimulated hearts in the same blots (n = 3, dashed line). * p<0.05 Iso vs. respective Basal, t test. (TIF) [file pone.0160813.s002.tif]
